# Polypi of Middle Ear and Auditory Canal as a Cause of Chronic Supurative Otitis

**Published:** 1891-12

**Authors:** R. C. Hodges

**Affiliations:** Galveston, Lecturer on Diseases of the Eye, Med. Dep’t Univ. Texas; Chairman Section on Opthalmology and Otology Texas State Medical Society


### DANIEL'S TEXAS MEDICAL JOURNAL.

A Representative Organ of the Medical Profession, and an Exponent of Rational Medicine; devoted to the Organization, Advancement and Elevation of the Profession in Texas.

PUBLISHED MONTHLY.—SUBSCRIPTION $2.00 A YEAR.

Vol. VII. AUSTIN, DECEMBER, 1891. No. 6.

### Original Articles.

CONTRIBUTED EXCLUSIVELY TO THIS JOURNAL.

*The Articles in this Department are accepted and published with the understanding that we are not responsible for, nor do we indorse the views and opinions of the writers by so doing.*

---

For Daniel's Texas Medical Journal.

### POLYPI OF MIDDLE EAR AND AUDITORY CANAL AS A CAUSE OF CHRONIC SUPPURATIVE OTITIS.

BY R. C. HODGES, M. D., GALVESTON,

Lecturer on Diseases of the Eye, Med. Dep't Univ. Texas; Chairman Section on Opthalmology and Otology Texas State Medical Society.

CASES FROM PRACTICE.

ONE of the most common causes of persistent chronic suppurative otitis is the presence of polypi or polypoid granulations in the tympanic cavity and auditory canal. There are other conditions causing this disease, but it is my purpose in these notes to call especial attention to this particular class of cases. The condition is common to all ages up to middle life, more frequent in children and young adults. There exists among the laity a most unreasonable and erroneous prejudice against stopping a discharge from the ear of a child or infant. This neglect of an acute trouble too often leads to the formation of these growths and to the various other conditions which cause chronic suppurative otitis. There exists also a prejudice among the medical profession against chronic ear troubles, which is due to the fact that they regard these diseases as among the most obstinate, and hesitate to engage in a contest which may last weeks, months or even years. Taking chronic ear troubles in general we will find that the cures average very well with any other class of chronic diseases, and when we isolate special forms of ear disease, we find some which yield more readily to treatment than other chronic troubles, and give brilliant results. This is especially true of those diseases due to polypoid growths of the middle ear and auditory canal. A careful examination will always reveal the presence of polypoid or granular growths. Not a mere inspection of the parts, for the eye will often be deceived.

After carefully cleansing the ear with absorbent cotton and a solution of peroxide of hydrogen (I prefer this method to the use of the syringe), I apply, with absorbent cotton on a probe, a ten per cent. solution of cocaine, and after ten minutes, carefully manipulate the parts to ascertain if the mem. tymp. is intact. Very frequently, but for this precaution, one would err in diagnosis, from the fact that in many cases these polypoid growths are so close together that the cotton probe will flatten or pack them in the bottom of the canal, so that the smooth, shining surface may be mistaken for the memb. tympani and the case diagnosed as a simple suppurative otitis. These growths bleed readily and are easily detached, and whenever I use a cotton probe and find blood stains, I involuntarily reach for my curette. I use the curette in preference to nitric acid or chromic acid or the galvano cautery, for the same reason that in the majority of cases I use the cold snare in nasal surgery. With the curette we obtain a clean, fresh wound which heals rapidly, whereas in using nitric acid, chromic acid or the cautery we cannot limit their effects to the growths to be destroyed, and we replace a chronic inflammatory process with an acute inflammation of an ugly character. A burn is even a more serious form of wound to treat in the ear than in any other part of the body, and frequently produces a condition more serious than the one we are working to relieve.

I herewith cite a few clinical cases, fifteen out of the last twenty-five cases of suppurative otitis I have treated, believing that one practical illustration is worth more than any number of abstract theories:

CASE I.—S. G., infant, age one year. Twelve months previous to consultation had suffered from an acute otitis of the left ear. The acute symptoms subsided in a reasonable time, but at intervals since the acute attack there has been a very offensive discharge from the ear. Wiping out the ear with absorbent cotton, I found the cotton discolored with blood. I first used a solution of hydrogen peroxide and then applied a ten per cent. solution of cocaine. (In this connection, I wish to remark that several authorities have claimed that cocaine is of doubtful value as a local anæsthetic in operations within the auditory canal. This is true with regard to acute cases, but I have found it of great value when curetting is necessary in chronic cases.) After ten minutes, I removed with a sharp ear curette, two polypi the size of rice grains, and after the slight bleeding was checked, applied a very small quantity of boracic acid in powder. No other treatment was used and the discharge was completely checked by this operation.

CASE 2.—Addie W., aged fourteen years. She had been troubled with offensive discharge from left ear for an indefinite period, covering several years. Examination revealed, apparently, an auditory canal somewhat shorter than normal, with a bulging, prominent memb. tymp. which proved on careful examination to be a large polypus, filling the entire lumen of the canal. This, I removed with Wilde's Snare, when another polypus was revealed, somewhat smaller than the first. This, I also removed, and repeated the process until I had removed no less than *twenty-three* separate and distinct polypi, from the size of a bean to the size of a grain of rice, considerable pus and epithelium, and found, to my surprise, the memb. tymp. intact and showing very little irritation. The result was very satisfactory, but little inflammation following, and in less than two weeks the case was dismissed with hearing distance normal, and there has been no evidence of any return of the growth or discharge.

CASE 3.—L. L., age five and a half years. Had had purulent discharge from right ear for three years. Found the canal filled with pus, very offensive in odor. Carefully cleaning the parts, I discovered a large perforation of memb. tymp., nearly two-thirds of which was destroyed. Springing from the middle ear and presenting through the opening were several polypi. These were removed after the usual applications, by means of curette. I found also a necrosed surface, which at a second operation under chloroform, was scraped smooth with a small bone curette. This operation had to be repeated several times, once on account of the re-formation of the growths. Necrosis of the osicles and localized necrosis of the bony canal is a frequent complication in these cases, the polypi springing from the sinus leading to the necrosed bone. Owing to the patient being very refractory, examinations and treatment were made with difficulty, and the case did not yield as readily as I had expected and hoped, but the offensive discharge finally ceased, and at my last examination I found the memb. tymp. *fully restored* and hearing normal. The result in this case was particularly satisfactory, as the patient had been under the treatment of many other physicians and by each in turn abandoned as hopeless.

CASE 4.—Mr. W. consulted me for chronic, offensive and very profuse discharge from right ear, which had existed for several years, and which was no doubt largely responsible for his exceedingly debilitated condition. He had occasional pains and frequent attacks of dizziness. I found the memb. tymp. almost destroyed, while springing from the attic and floor of the tympanic cavity and external to the memb. tymp. on the posterior inferior surface of the auditory canal were several polypi. In two operations I removed the growths with the curette. There was considerable hemorrhage which was easily controlled. The after treatment consisted of the local use, three times daily, of a solution of peroxide of hydrogen, tonics and generous diet, and in two weeks the memb. tymp. was restored to such an extent as to leave a well cicatrized perforation about the size of a pin head; hearing distance slightly reduced. The dizziness disappeared after the first operation, patient dismissed after two weeks, and in six weeks from date of first consultation he had increased 15 pounds in weight.

CASES 5 AND 6.—These were cases of chronic otitis, external, one a child of six years, the other an adult, with polypi of auditory canal. The polypi were removed, and the cases treated but a few days and dismissed, and there has been no return of the trouble.

CASE 7.—Mr. H., age forty-five years, gave history of chronic suppurative otitis of both ears. There were frequent acute manifestations. He consulted me during one of these acute attacks, and after it had subsided, I removed polypi from both tymp. cavities, the memb. tymp. being fully restored, the left presenting a large perforation. There has been a recurrence of the trouble in this ear. Owing to the fact of his being a commercial traveler, his visits were uncertain and irregular, and he was constantly exposed to all the changes of weather, habit and diet common to his occupation.

CASE 8.—Master G., age 15, had been treated for over four years by various physicians for discharge from left ear. This case illustrates how readily one could be misled in diagnosis by a hurried or superficial examination. At the first two visits I could detect no growths, the case appearing to be a simple chronic suppurative otitis of the middle ear with partial destruction of the memb. tymp. At his third visit I used an Eustachian catheter of large size, and applied through it the full force of compressed air, from a cylinder registering thirty-five pounds. After this, on examining the ear, I found, presenting in the opening in the memb. tymp., a long and slender polypus, with small pedicle, which I easily grasped and removed with angular forceps. In three weeks this patient was dismissed. The discharge was perfectly cured, but the perforation remained. Two slight attacks of acute inflammation have occurred since, due to exposure after bathing.

The remainder of the cases, with the exception of fourteen and fifteen present no features of special interest; there was no perforation of the memb. tymp., and no necrosis. All of these four patients were adults, two of them under treatment for one week, one for three weeks, and one for four weeks.

CASE 14.—Mrs. M. consulted me on account of pain in the ear, dizziness and profuse discharge. On account of having endured so much in the treatment, she was a little disinclined to submit to further treatment until forced by the condition of the ear to do so. I found in the external auditory canal, dependent from the upper portion, and very close to the memb. tymp. a polypus, broad and flat, which could be raised so as to expose the memb. tymp. The polypus was easily removed with the curette, and at the point of attachment I found necrosed bone. This I curetted, using as little force as possible, always having in view in these cases the danger of too free manipulation of the parts. Despite these precautions there was considerable reaction, severe inflammation, very copious discharge, swelling of canal, quite marked mastoid tenderness and redness of skin over mastoid region. This lasted four or five days, but yielded to the free use of hot water and hydrogen peroxide locally, and internal treatment by antipyretics and anodynes. The discharge has entirely ceased and the result is perfect.

CASE 15.—Mr. E. C., had for over a year been annoyed by a discharge from the right ear, at times slight, at others profuse. At time of consultation, there was a profuse, acrid, offensive discharge, the external ear showing an ugly, eczematous excoriation. From the external auditory canal I removed one of the largest polyps I have encountered. It nearly filled the auditory canal. The treatment was limited to this one operation, and the free washing of the canal with hydrogen peroxide and the application of a one per cent. solution of Fuchsine in collodion. There has been no discharge since, and the external inflammation has almost entirely disappeared, and the hearing is normal.

In these few notes from practice I have necessarily been brief and to the point, and have carefully avoided touching on other forms of suppurative otitis, as my intention, stated at the beginning of this paper, is to call special attention to this form of chronic disease of the ear, and to demonstrate by cases cited how brilliant and satisfactory the results are when proper treatment is applied.

Eleven out of the fifteen cases had rung the changes on iodoform, iodol, creolin, bichloride mercury, listerine, etc., etc. They demonstrate the necessity of exercising all possible vigilance in examinations even of apparently simple cases. Given a clear diagnosis, followed by proper treatment, a cure should result.

No other class of non-malignant diseases cause a greater drain upon the vital powers than these suppurative diseases of the ear, no matter what the cause. Infants and children regain color and strength, with the rest and improved nutrition following a cure ; adults escape from a most dangerous condition that too often leads to deafness, paralysis, abscess of the brain, and death. Therefore there should be no question as to these cases being worthy of our best efforts.

